# Distribution of organic and inorganic mercury in the tissues and organs of fish from the southern Baltic Sea

**DOI:** 10.1007/s11356-018-3336-9

**Published:** 2018-10-04

**Authors:** Lucyna Polak-Juszczak

**Affiliations:** Department of Food and Environmental Chemistry, National Marine Fisheries Research Institute, ul. Kollataja 1, 81-332 Gdynia, Poland

**Keywords:** Fish, Mercury, Bioaccumulation, Biomagnification, Nutrition, Trophic chain

## Abstract

The aim of this study was to determine the distribution of total mercury (THg), methylmercury (CH_3_Hg^+^), and inorganic mercury (Hg_inorg_) in the tissues and organs of fish depending on species, tissue, and organ, and their bioaccumulation in tissues and biomagnification throughout the trophic web. The study included four species of fish (herring, sprat, cod, and eel) from the southern Baltic Sea. The concentrations of the different forms of mercury were determined in tissues and internal organs. Intra-specific differences in levels of THg, CH_3_Hg^+^, and Hg_inorg_ in the tissues and organs were determined. Muscle contained the highest proportions of THg and CH_3_Hg^+^ in comparison to that in the internal organs. Differences in concentrations of THg, CH_3_Hg^+^, and Hg_inorg_ in the tissues and organs of fish were related to their preferred prey. The bioaccumulation of CH_3_Hg+ in the tissues and organs of predatory fish at the highest trophic levels was greater than in the liver and digestive tract of fish species at lower trophic levels, in which Hg_inorg_ predominated. The high concentrations of CH_3_Hg^+^ in eel and cod and the low levels in herring and sprat were linked with their food and the transfer of this element among species. The results suggested that the type of food, feeding habits, and trophic position were important parameters that influenced the transfer and biomagnification of mercury in fish.

## Introduction

Mercury is recognized as a major environmental pollutant and a hazardous metal for living organisms. Mercury bioaccumulation in the aquatic food web extends from its base of microorganisms and plankton to predatory fish and mammals at the top of the food chain. Fish take up mercury by absorbing it through the body surface and gills, but the primary source is the diet (Hall et al. 1997; Jaeger et al. 2009). Feeding habits of fish species (piscivorous, omnivorous, non-piscivorous) impact mercury levels in tissues and organs (Garcia et al. 2000; Berntssen et al. 2003). Mercury taken up from the water through the gills and skin is primarily Hg_inorg_ since this form dominates in water and sediments. Inorganic mercury contained in the sediments is transformed by microbes such as sulfate- and iron-reducing bacteria into the organic form of methylmercury (Flemming et al. 2006). All forms of mercury are bioaccumulated in the tissues and organs of fish (Barwick and Maher 2003; Leaner and Mason 2004), while methylmercury also biomagnifies the trophic chain (Kehrig et al. 2010). Organic mercury usually dominates in the muscles of fish, and concentrations are usually significantly correlated with fish age and weight (Polak-Juszczak 2017). Methylmercury is the strongest of the neurotoxins, and it is mutagenic and causes disruptions in the circulatory system, the nervous system, reproductive parameters, and spawning (Hammerschmidt et al. 2002; Kwaśniak and Falkowska 2012). Changes in biochemical processes in fish occur at concentrations of CH_3_Hg^+^ from 0.5 to 1.2 μg g^−1^ w.w. in dorsal muscle (Dietz et al. 2010). High concentrations of THg (range of approximately 0.1 to 1.0 μg g^−1^ w.w.) in liver and dorsal muscle lower the condition coefficient of fish (Cizdziel et al. 2003). The majority of studies focusing on mercury concentrations in fish refer to the muscles of freshwater fish. Significantly, fewer data are available on the concentrations of CH_3_Hg^+^ in marine fish, and the available literature reports levels of this element mainly for muscles. There is a lack of data concerning concentrations of CH_3_Hg^+^ in other tissues (skin, gills) and organs (liver, kidney, heart, spleen, digestive tract) of marine fish. The present study is the first to examine the different forms of mercury in the tissues and organs of fish from the southern Baltic Sea (eel, cod, herring, sprat) that have different feeding strategies (phytophagous, benthophagous, predatory) and semiaquatic carnivores, including piscivores and omnivores.

The aim of this study was to evaluate the following: (1) differences in concentrations of mercury forms (THg, CH_3_Hg^+^, and Hg_inorg_) among species (eel, cod, herring, sprat); (2) intra-specific correlations among concentrations of THg, CH_3_Hg^+^, and Hg_inorg_ in tissues (muscle, skin, gills) and organs (liver, kidney, heart, spleen); (3) bioaccumulation as a function of the diet (phytophagous, benthophagous, predatory, carnivorous, omnivorous) and biomagnification along the food chain.

## Materials and methods

### Materials

The study material comprised four fish species that occur most commonly in the Baltic Sea, i.e., cod (*Gadus morhua*), eel (*Anguilla anguilla*), herring (*Clupea harengus*), and sprat (*Sprattus sprattus*). The fish were caught in the Polish coastal zone of the southern Baltic Sea (central and eastern Baltic region) in fall and winter 2016 after spawning. The species selected do not have the same diets and are carnivores (cod), omnivores (eel), and herbivores (herring, sprat). Samples of muscle tissues, skin, gills, and internal organs (liver, heart, spleen, digestive tract, and gonads) were collected from each 1specimen. The muscles analyzed were homogenized, while whole organs were analyzed. THg and CH_3_Hg^+^ assays were performed on the tissues (muscle, skin, gills) and organs (liver, spleen, heart, digestive tract) of cod and eel. Mercury analysis of herring was performed for muscles, the liver, gills, and the digestive tract, while that of sprat was performed for gills, the digestive tract, and for whole individuals. THg and CH_3_Hg^+^ assays were performed on the tissues and organs of ten individuals of each species.

### Analysis of total mercury concentration

Total mercury content was assayed with the cold vapor atomic absorption method in an AMA 254 mercury analyzer. The analyses were conducted according to the following procedure. Tissue samples of about 100 mg were placed in the combustion chamber of the analyzer where they were dried and then burned at a temperature of 600 °C in an oxygen atmosphere. The measurements were conducted as follows: fish muscle tissue, drying time 65 s; decomposition time 160 s; waiting time 60 s; fish liver tissue, drying time 100 s; decomposition time 160 s; waiting time 60 s. Each series of analyses was preceded by measurements of mercury in reference materials of a similar matrix.

### Analysis of methylmercury concentration

Methylmercury content was determined according to the method described by Maggi et al. (2009) and Tong et al. (2012). In brief, the procedure was as follows: from 1 to 2.0 g of homogenized fish tissue was weighed out and placed in 50-ml test tubes for centrifugation; 5 ml of hydrochloric acid (18% *v*/*v*) and 5 ml of toluene were added; the test tubes were placed in an ultrasound water bath for 30 min; then the test tubes were centrifuged for 30 min at 3500 rev/min. The toluene layer was moved to 10-ml test tubes, while 5 ml of toluene was added to the remaining solution in the test tubes, which were again placed in an ultrasound bath for 30 min, after which they were centrifuged again, as described above. After centrifugation, the upper layer of toluene was separated and added to the previously separated toluene, and 1 ml of cysteine hydrochloride solution (1% cysteine hydrochloride solution in a 20% sodium citrate solution) was added to the combined toluene layers, and this was placed in an ultrasound bath for 30 min, and then centrifuged again, as described above. Before measurements, the upper layer of toluene was removed with a syringe. The samples (100 μL) were placed in the combustion chamber of the AMA 254 analyzer where they were dried and then burned at a temperature of 600 °C in an oxygen atmosphere. The measurements were conducted according to the following procedures: drying time 70 s, decomposition time 120 s, and waiting time 50 s. The same procedure was used with a blank sample. Each series was preceded by measuring the form of mercury and methylmercury in the certified reference material. The concentrations of Hg_inorg_ in fish tissues were calculated as the differences between concentrations of THg and CH_3_Hg^+^.

### Measurement quality control

The accuracy of the chemical analysis was verified using reference material before every measurement series. The following materials were used for THg: TORT-2 lobster hepatopancreas (National Research Council of Canada) at a concentration of THg 0.27 ± 0.06 mg kg^−1^ and BCR-422 cod muscle (Joint Research Centre Institute for Materials and Measurements, Geel, Belgium) with a concentration of THg of 0.559 ± 0.016 mg kg^−1^. The following reference material was used for measurements of MeHg: TORT-2 lobster hepatopancreas with a concentration of MeHg 0.152 ± 0.013 mg kg^−1^ and BCR-463 tuna fish (Joint Research Centre Institute for Materials and Measurements, Geel, Belgium) with a concentration of MeHg 3.04 ± 0.16 mg kg^−1^. The recovery ranges were from 90 to 110%. During validation, the method detection limit (MDLs) for THg was 0.5 μg kg^−1^ and for MeHg, it was 5 μg kg^−1^. All samples were analyzed in duplicate. The results presented are arithmetic averages with a standard deviation of less than 10%.

### Statistical analysis

Statistica 8.0 software was used to perform descriptive statistics and regression analysis for all data. The mercury and methylmercury concentrations were tested for normality (Shapiro-Wilk test). Statistically significant differences in concentrations of THg, CH_3_Hg^+^, and CH_3_Hg^+^ percentages were determined using parametric (ANOVA) or non-parametric (Kruskal-Wallis) tests. Relationships between THg and CH_3_Hg^+^ concentrations in the tissues and organs of fish (non-parametric data) were tested using Spearman’s rank correlation test. Analysis of differences in concentrations THg, CH_3_Hg^+^, and Hg_inorg_ among the different ecological groups of fish (carnivorous, omnivorous, and herbivorous) was analyzed statistically with one-way ANOVA and Tukey’s post-hoc test. All null hypotheses were tested at a significance level of *p* < 0.05.

## Results and discussion

The paper presents the measurements of THg, CH_3_Hg^+^, and Hg_inorg_ concentrations in the tissues and organs of four fish species from the Baltic Sea. The article describes mercury bioaccumulation in fish tissues and organs and mercury biomagnification in the food chain.

### Distribution of THg, CH_3_Hg^+^, and Hg_inorg_ in the tissues and organs of fish and bioaccumulation

Metal accumulation in fish depends primarily on metal concentrations in ambient water, prey, and habitats (Kojadinovic et al. 2007). Mercury accumulates in muscle tissues mainly in predatory fish species, which means that muscle tissue is a suitable monitor of environmental mercury. In marine and freshwater ecosystems, mercury can be transferred to higher levels of the trophic pyramid through biomagnification (Hosseini et al. 2013). Mercury concentrations were analyzed in four fish species that occupy different levels of the trophic chain. Cod, as a predator, like eel, occupies the highest trophic level, while herring and sprat as planktonophages are on a lower trophic level. The results of the study indicate that there are significant differences in mercury concentrations depending on fish species, tissue, and organs (Tables 1 and 2). High concentrations of THg occurred in eel muscles and liver (Table 1). Data on the tendencies for mercury to accumulate in the muscles and liver have been published by many researchers, including Mc Intyre and Beauchamp (2007), Halvelková et al. (2008), Vieira et al. (2011), and Bergés-Tiznado et al. (2015). Similar THg concentration gradients, which were also at a high level, were determined in the tissues and organs of cod, and higher THg concentrations were found in muscle. High levels were also found in the heart and spleen, while significantly lower levels were found in the digestive tract, gills, and skin. The lowest concentrations, which exhibited the reverse gradient, were for those of THg in the tissues and organs of herring and sprat (Table 2). The highest concentrations of THg were in the herring liver, and these were significantly lower than those in the muscle and the digestive tract. Among the fish species assayed, THg occurred at the lowest concentrations in sprat. The high levels of THg in the digestive tracts of herring and sprat in comparison to those in other tissues, which were noted simultaneously with a low share of CH_3_Hg^+^ in this organ, are noteworthy (Table 2). The concentrations gradations of THg in the tissues and organs of the fish species studied were as follows:

- eel – muscle > liver > heart > spleen > digestive tract > gills > skin
- cod – heart > muscle > spleen > liver > digestive tract > gills > skin
- herring – liver > digestive tract > muscle > gills
- sprat – digestive tract > whole specimen > muscle > gills

**Table 1:** Biometric fish data, concentrations of THg, CH_3_Hg^+^, and Hg_inorg_ (mean, standard deviation, range) and the share of CH_3_Hg^+^ and Hg_inorg_ in the THg in tissues and organs of cod and eel from the southern Baltic Sea

	*N*	Biometric data		Tissues/organs						
				Muscles	Liver	Skin	Gills	Heart	Digestive track	Spleen
		TL [cm]	TW [g]	THg [μg kg^−1^ wet wt.]						
Cod	10	44.3 ± 5.8 38.2–56.1	941 ± 451 438–2027	64.1 ± 25.1 46.4–136.1	24.1 ± 24.7 7.6–37.1	9.6 ± 9.2 1.1–31.4	17.2 ± 8.1 9.1–29.5	73.9 ± 52.8 19.5–140.1	20.1 ± 8.5 6.2–36.5	41.8 ± 19.3 16.3–82.1
Eel	11	74.6 ± 8.5 64.3–92.1	868 ± 287 545–1530	232.0 ± 120 57.2–386.1	193.2 ± 149.3 21.3–440.2	7.1 ± 4.5 2.5–15.2	39.9 ± 23.9 12.5–58.5	120.1 ± 89. 19.5–281.6	49.1 ± 34.8 17.3–92.1	79.7 ± 67.2 14.1–179.2
	N	TL [cm]	TW [g]	CH_3_Hg^+^ [μg kg^−1^ wet wt.] (% CH_3_Hg^+^)						
Cod	10	44.3 ± 5.8 38.3–56.4	941 ± 451 438–2027	53.8 ± 21.7 35.5–115.5 (83.9)	18.1 ± 16.7 2.9–35.5 (75.1)	8.2 ± 5.4 1.9–19.9 (85.4)	9.9 ± 6.4 3.0–21.8 (57.6)	19.4 ± 12.1 7.0–49.7 (26.2)	15.2 ± 7.3 4.0–26.1 (75.6)	17.2 ± 9.9 4.5–34.7 (41.1)
Eel	11	74.6 ± 8.5 64.2–92	868 ± 287 545–1530	178.8 ± 113 42.1–378.2 (77.1)	160.6 ± 131.7 18.2–397.2 (83.1)	5.9 ± 5.6 1.1–14.4 (83.1)	34.7 ± 18.7 14.2–58.0 (86.9)	84.5 ± 77.9 13.1–258.5 (70.4)	35.4 ± 23.8 7.9–77.3 (72.1)	35.3 ± 33.4 8.1–118.5 (44.3)
	N	TL [cm]	TW [g]	Hg_inorg_ [μg kg^−1^ wet wt.] (% Hg_inorg_)						
Cod	10	44.3 ± 5.8 38.3–56.4	941 ± 451 438–2027	10.3 ± 7.1 4.5–24.3 (16.1)	6.0 ± 9.4 1–31.5 (24.9)	1.4 ± 2.7 0.6–11.5 (14.6)	7.3 ± 3.2 4.3–12.5 (42.4)	54.5 ± 42.7 3.3–108.0 (73.7)	4.9 ± 3.2 1.8–10.5 (24.4)	24.6 ± 16.8 10.8–69.0 (58.9)
Eel	11	74.6 ± 8.5 64.2–92	868 ± 287 545–1530	53.2 ± 42.5 8.1–148.1 (22.9)	32.6 ± 37.8 2.1–129.7 (16.9)	1.2 ± 1.7 0.8–5.7 (16.8)	5.2 ± 6.02 0.1–10.9 (13.0)	35.6 ± 52.5 4.1–163.4 (29.6)	13.7 ± 15.1 3.5–14.6 (27.9)	44.4 ± 42.2 6.0–119.4 (55.7)

*TL* total length, *TW* total weight

**Table 2:** Biometric fish data, concentrations of THg, CH_3_Hg^+^, and Hg_inorg_ (mean, standard deviation, range) and the share of CH_3_Hg^+^ and Hg_inorg_ in the THg in the tissues and organs of herring and sprat from the southern Baltic Sea

Species	*N*	Biometric data		Tissues/organs			
				Muscles	Liver	Gills	Digestive track
		TL [cm]	TW [g]	THg [μg kg^−1^ wet wt.]			
Herring	10	19.6 ± 3.1 15.2–23.1	56.2 ± 21.3 391.2–78.3	18.6 ± 9.4 8.2–39.3	37.4 ± 20.8 11.6–90.1	9.7 ± 5.5 4.6–20.7	29.6 ± 13.4 13.6–55.2
Sprat	10	12.3 ± 2.3 7.0–19.2	14.7 ± 4.9 9.1–17.7	10.0 ± 4.6 2.1–16.9	10.8 ± 5.2a 2.3–17.8	6.5 ± 2.6 3.0–9.7	15.4 ± 7.9 7.0–25.6
	N	TL [cm]	TW [g]	CH_3_Hg^+^ [μg kg^−1^ wet wt.] (% CH_3_Hg^+^)			
Herring	10	19.6 ± 2.0 15.1–23.2	56.2 ± 21.3 391.1–78.3	14.8 ± 9.2 4.4–31.6 (79.6)	10.1 ± 5.9 5.7–22.2 (27.0)	3.7 ± 2.8 1.6–10.6 (38.1)	4.8 ± 3.2 1.5–11.3 (16.2)
Sprat	10	12.3 ± 2.3 7.2–19.1	14.7 ± 4.9 9.1–17.7	6.3 ± 2.8 1.8–10.2 (63.0)	4.8 ± 2.1a 1.7–7.2 (44.4)	2.9 ± 1.4 1.0–4.1 (44.6)	3.5 ± 1.6 0.8–5.8 (22.7)
	N	TL [cm]	TW [g]	Hg_inorg_ [μg kg^−1^ wet wt.] (% Hg_inorg_)			
Herring	10	19.6 ± 2.0 15.1–23.2	56.2 ± 21.3 391.1–78.3	3.8 ± 2.1 1.2–7.7 (20.4)	27.3 ± 17.0 5.9–56.3 (73.0)	6.0 ± 4.6 2.7–13.9 (61.9)	24.8 ± 11.1 12.1–43.9 (83.8)
Sprat	10	12.3 ± 2.3 7.2–19.1	14.7 ± 4.9 9.1–17.7	3.7 ± 2.2 0.3–6.9 (37.0)	6.0 ± 4.4a 0.6–12.5 (55.6)	3.6 ± 1.7 1.4–6.2 (55.4)	11.9 ± 5.4 5.2–17.6 (77.3)

^a^Whole individuals

The dependencies indicate differences in THg concentrations in the tissues and organs of the species. Significant differences were also noted in CH_3_Hg^+^ concentrations in the tissues and organs of fish. Muscle contained the highest amount of organic mercury. The CH_3_Hg^+^ concentration gradients in the tissues and organs of the fish species studied were as follows:

- cod – muscle > heart > liver > spleen > digestive tract > gills > skin
- eel – muscle > liver > heart > digestive tract > spleen > gills > skin
- herring – muscle > liver > digestive tract >gills
- sprat – muscle > whole specimens > digestive tract > gills

High CH_3_Hg^+^ levels in fish muscles are the result of the high affinity that this element has for thiol groups in amino acids that are one of the components of protein, for example cysteine (Zhang and Planas 1994; Ruelas-Inzunza et al. 2003; Leaner and Mason 2004). Further, CH_3_Hg^+^ is characterized by its high bioavailability, which means that it is almost completely assimilated by fish. The organic form of mercury dominated in the muscle of all of the fish species assayed, although at different levels (Tables 1 and 2). The present study confirmed the high share of CH_3_Hg^+^ in the THg in the muscle of cod (above 80%), eel (77%), herring (79%), and sprat (63%) (Tables 1 and 2). The share of CH_3_Hg^+^ in the THg in the liver, digestive tract, gills, and skin of cod and eel exceeded 70%, while that in the herring liver was 27.8%. The share of CH_3_Hg^+^ in the livers of the fish species was linked with different methylmercury detoxification strategies that are related fundamentally to the direct elimination and/or biotransformation of methylmercury (Kehrig et al. 2009, 2010).

Concentrations of THg in fish from the southern Baltic region are shown in Tables 1 and 2. Concentrations of THg in cod and herring from other regions of the open Baltic Sea in 2011–2016 period exceeded the threshold value of 20 μg kg^−1^ w.w. in all areas assessed, except the Arkona Basin and some of the Danish and Swedish coastal areas. Our research confirms the preceding data. The THg content in cod from the southern Baltic is 64 μg kg^−1^, while the THg level in herring from our research area is also below 20 μg kg^−1^ (Tables 1 and 2). The lowest mean concentration of THg in the Arkona Basin was 17.6 μg kg^−1^ w.w. In the Gulf of Finland and the Kattegat, THg concentrations in fish muscles were similar at 34.5 and 36.5 μg kg^−1^ w.w. The highest value of 58.0 μg kg^−1^ w.w. was found in the Gdansk Basin. No temporal changes in THg concentrations in fish from the Baltic Sea were detected (HELCOM 2017; Boalt et al. 2014). Concentrations of THg in the muscles of fish from different regions of the Baltic Sea were as follows (μg kg^−1^ w.w.): Sweden 97.9 (29–260); Germany 101.3 (86.7–124.3); Denmark 17.3–33.6; Poland 42–104; Latvia/Estonia, Gulf of Riga 40; and Finland, Gulf of Finland; 60–100 (Hedman et al. 2011).

In aquatic environments, the predominant mercury species is Hg_inorg_, and it readily undergoes biochemical transformation (Wang and Wong 2003). CH_3_Hg^+^ is formed during methylation with the participation of bacteria, and this form enters the aquatic trophic chain where concentrations increase in subsequent links (He et al. 2007). Fish adsorb Hg_inorg_ directly from the environment through the gills and skin. This element occurred at low levels in cod and eel skin (approximately 15% of THg), while cod gills contained higher levels (up to 42% of THg). High levels of Hg_inorg_ were detected in the heart and spleen of cod (74 and 58% of THg), which indicated that CH_3_Hg^+^ demethylation to its inorganic form was intense in these cod organs. Many researchers confirm the phenomenon of CH_3_Hg^+^ demethylation in the internal organs of fish (Palmisano et al. 1995; Watras et al. 1998; Zhang et al. 2001; Storelli and Marcotrigiano 2002; Gonzalez et al. 2005; Halvelková et al. 2008; Ostertag et al. 2013; Rodríguez Martín-Doimeadios et al. 2014). Demethylation does not occur in fish muscle. Cod and eel muscle contained high levels of CH_3_Hg^+^ while Hg_inorg_ comprised approximately 15% of THg, which confirmed the lack or weakness of demethylation. The levels of mercury forms in herring and sprat contrasted those in cod and eel. Inorganic mercury occurred at high levels in gills (approximately 60% of THg), which suggested that this element was taken up directly from the water (Table 1). High Hg_inorg_ concentrations in the digestive tracts of sprat and herring (approximately 80% of THg) indicated that this mercury form was ingested with food (Table 2). The majority of the food of these species is plankton, which contains substantial quantities of Hg_inorg_ (Beldowska et al. 2015). High concentrations of inorganic mercury in the herring liver (73% of THg) resulted from the accumulation of mercury ingested with food, which was confirmed by high Hg_inorg_ shares in THg (Table 2) and high Rs coefficients (Table 3).

**Table 3:** Relationship among concentrations of THg, CH_3_Hg^+^, and Hg_inorg_ in tissues and organs of fish from the southern Baltic Sea

Tissue/organ	Cod	Eel	Herring	Sprat	Cod	Eel	Herring	Sprat	Cod	Eel	Herring	Sprat
	THg/CH_3_Hg^+^				THg/Hg_inorg_				CH_3_Hg^+^/Hg_inorg_			
Muscles	0.645	0.718	0.854	0.963	*	*	*	0.867	*	*	*	0.758
Liver	0.644	0.973	0.656	0,794^a^	0.720	0.721	0.988	0.964^a^	*	0.685	0.644	0.759
Skin	0.706	0.682	–	–	0.744	0.795	–	–	*	*	–	–
Gills	0.917	0.964	0.733	0.667	0.666	*	0.867	0.717	*	*	*	*
Heart	*	0.818	–	–	0.879	0.783	–	–	*	*	–	–
Digestive track	0.952	0.954	0.781	0.683	0.638	0.727	0.939	0.883	0.644	0.633	0.669	.667
Spleen	0.794	0.917	–	–	0.782	0.917	–	–	*	0.733	–	–

*p > 0.05 no dependency; ^a^Whole individuals; – no data

### Correlation among concentrations of THg, CH_3_Hg^+^, and Hg_inorg_ in fish tissues and organs

A positive correlation was identified in cod between THg and CH_3_Hg^+^ concentrations in tissues (muscles, gills, and skin) and organs (liver, digestive tract, and spleen) at Rs correlation coefficients ranging from 0.644 to 0.952 (Table 4). High positive correlations were also confirmed between concentrations of THg and CH_3_Hg^+^ in the tissues and organs of eel at high Rs correlation coefficients of 0.682 to 0.973. The strong correlations between these forms of mercury in the digestive tract indicated that cod and eel ingested CH_3_Hg^+^ with food, which was also indicated by the high share of this element in the digestive tract (cod 76%, eel 72% of THg). Ingested CH_3_Hg^+^ is transported through the intestinal mucosa to the blood and then into the organs where it is accumulated, mainly in the liver and muscle. CH_3_Hg^+^ intake from the environment is also reflected in high mercury levels in external organs, such as the gills and skin (Coelho et al. 2008; Pethybridge et al. 2010). Strong correlations of these forms of mercury occur in the gills of cod and eel, which indicates CH_3_Hg^+^ uptake from the environment. Concentrations of THg and CH_3_Hg^+^ in herring and sprat tissues (muscles and gills) and organs (liver and digestive tract) were positively correlated, but at lower Rs correlation coefficients (0.656–0.854). Weak correlations between THg/CH_3_Hg^+^ in herring and sprat digestive tracts and gills indicated a smaller intake of organic mercury with food and from the environment. These fish feed on phytoplankton and zooplankton, in which Hg_inorg_ dominated. Herring and sprat ingests Hg_inorg_ with food and water, which is confirmed by the high share of this form of mercury in the digestive tract (about 80% THg) and gills (over 55% THg). Hg_inorg_ ingested with food and water accumulates mainly in the liver, which is confirmed by strong correlations between THg/Hg_inorg_ in the livers of herring and sprat. The liver also plays a key role in detoxifying CH_3_Hg^+^, which is demethylated in this organ to less toxic form of Hg_inorg_ (Gonzalez et al. 2005; Storelli and Marcotrigiano 2002). High values of the Rs correlation coefficient for THg/Hg_inorg_ were noted in herring and sprat, while lower values were recorded for cod and eel, which mean that cods and eel ingest small amounts of Hg_inorg_ from the environment, and this form found in the liver is probably from the demethylation of CH_3_Hg^+^. No correlations between CH_3_Hg^+^/Hg_inorg_ were found in most of the tissues and organs of the studied fish species, which probably stemmed from high variations in the concentrations of these forms of mercury.

**Table 4:** Values of coefficient L:M (L = Hg_inorg_ in the liver; M = CH_3_Hg^+^ in the muscle) in southern Baltic Sea fish species with different nutritional habits

Species	L/M (*x* ± sd)	L/M range	Nutrition
Cod	0.117 ± 0.1	0–0.27	Piscivorous
Eel	0.195 ± 0.18	0.02–0.49	Omnivorous
Herring	2.771 ± 2.49	0.85–9,51	Herbivores
Sprat	0.935 ± 0.38	0.33–1.43	Herbivores

The analysis of fish from different trophic levels can provide information about the bioaccumulation and biomagnification that occurs even if the different species do not prey directly on each other (Kehrig et al. 2009). The variability of mercury in different fish species reflects dietary differences, and feeding habits are the most influential factors on the bioaccumulation and biomagnification of different forms of mercury in fish.

### Influence of diet and biomagnification in the food chain

Accumulation through the food chain is one of the main routes of mercury bioaccumulation in marine organisms (Mason et al. 2000; Barwick and Maher 2003). All mercury forms can bioaccumulate, but additional CH_3_Hg^+^ biomagnification to varying degrees in aquatic food chains depends on the amounts and forms of mercury. Biomagnification is associated with feeding habits and the trophic position from which food items originate (Turner and Swick 1983). Consumers on higher trophic levels, such as piscivorous fish, have higher mercury concentrations than organisms that feed at lower levels, for example, species that feed on benthic diatoms or invertebrates (Varanasi et al. 1994). The type of food marine organisms consume greatly influences the amount of mercury they assimilate, and there are marked differences among the concentrations of this element in microplankton, mesozooplankton, and fish. Mercury analysis of fish from different trophic levels can provide information on the processes of bioaccumulation and biomagnification that can occur even if the selected species do not feed directly on one another (Kehrig et al. 2010).

**Fig. 1:**
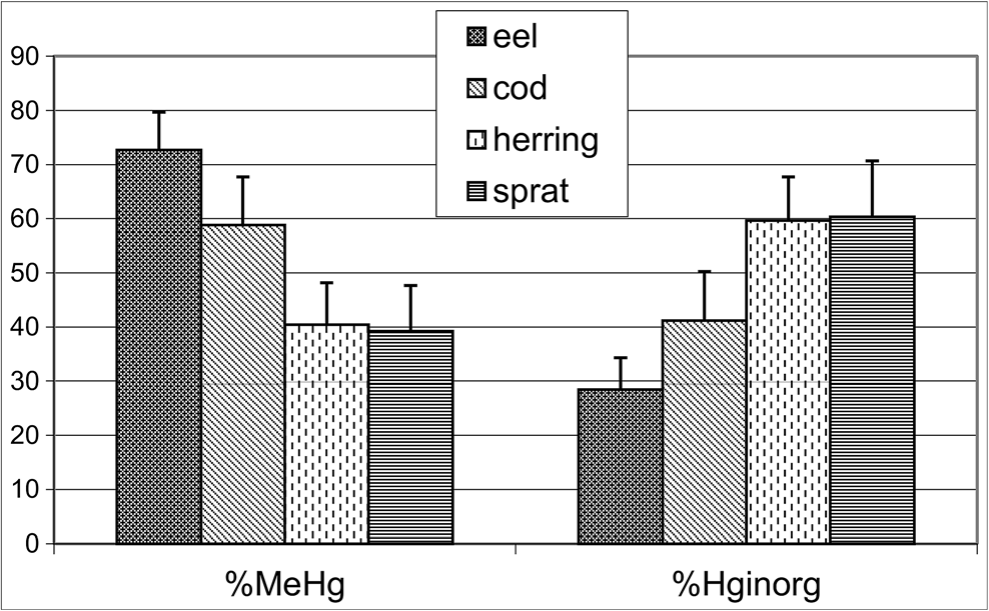
Percentage (%) of CH_3_Hg^+^ and Hg_inorg_ in the THg in gills of cod, eel, herring, and sprat from the southern Baltic Sea

**Fig. 2:**
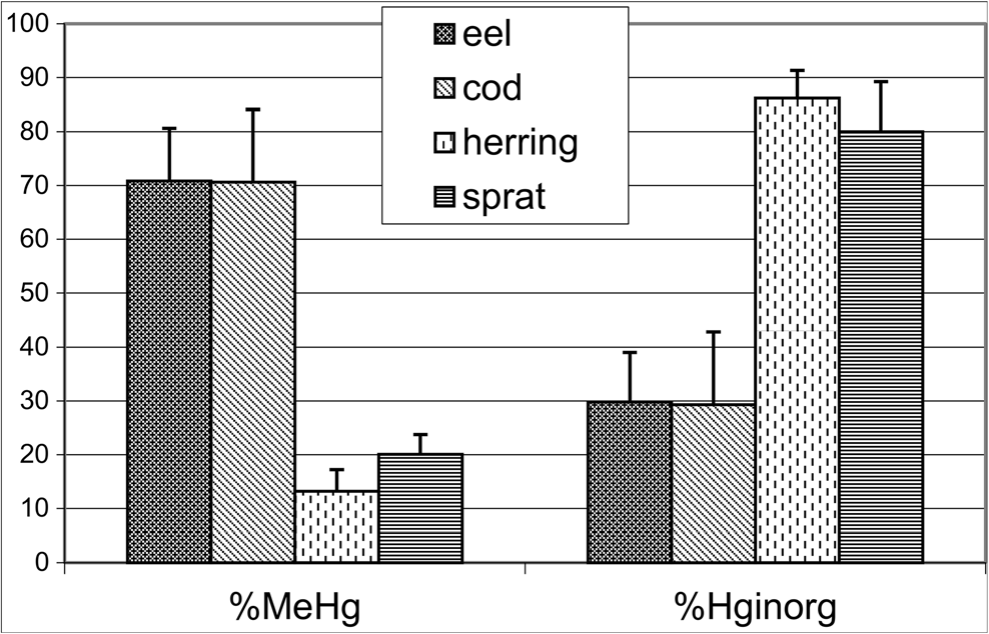
Percentage (%) of CH_3_Hg^+^ and Hg_inorg_ in the THg in gastrointestinal tract of cod, eel, herring, and sprat from the southern Baltic Sea

The results presented in this paper come from assays to determine THg, CH_3_Hg^+^, and Hg_inorg_ levels in four fish species (eel, cod, herring, sprat) from different levels of the trophic chain, with different feeding habits. Eel feed primarily on benthic fauna and small fish, but they can also consume prey of relatively large sizes. Additionally, mercury accumulates in sediments, and benthic organisms assimilate this element. Cod are predators that feed mainly on fish from the families *Clupeidae*, cod fry, and benthic crustaceans, and they are even cannibalistic. Herring feed on phytoplankton and zooplankton, small crustaceans and larval fish, while sprat feed on phytoplankton (Casini et al. 2004; Dziaduch 2011; Beldowska et al. 2015). The highest concentrations of mercury were confirmed in eel and cod tissues and organs, and CH_3_Hg^+^ dominated in the muscle and liver, but it also occurred at high levels in other internal organs (Table 1). High CH_3_Hg^+^ levels in eel and cod digestive tracts and gills indicated that these fish ingested this element with food, and they also absorbed it directly from the environment (Figs. 1 and 2). The gills are the first organ exposed to mercury suspended in water molecules and sediments. High CH_3_Hg^+^ concentrations in eel and cod gills (Fig. 1) indicated that the source were bottom sediments and the water (Beldowski et al. 2014). Abdolvand et al. (2014) attribute the high methylmercury percentages (> 90%) and concentrations observed in the gills of eel, a benthic species, to the influence of sediments. According to Watras et al. (1998), in fish from higher trophic levels, such as eel and cod, CH_3_Hg^+^ concentrations reflect the ingestion of this element with food that comprises organisms from lower trophic levels. While fish from higher positions on the trophic chain, such as cod and eel, mainly assimilate mercury as CH_3_Hg^+^ through food, this organic form of mercury represents only a small fraction (approximately 20%) of THg in herring and sprat tissues and organs (Fig. 2). Significantly more Hg_inorg_ occurred in the digestive tract of herring and sprat (80% of THg) than did in cod and eel.

The increase in CH_3_Hg^+^ concentrations in the food web indicates the transfer of CH_3_Hg^+^ from microplankton (from a lower level of the trophic chain) to a mesoplankton (to a higher level), and then up to predatory fish at the top of the trophic chain (Kehrig et al. 2010).

Our data showed that CH_3_Hg^+^ increased as the position of the fish species increased along trophic levels in the Baltic Sea: eel, cod > herring > sprat.

The results of our study indicated that Hg_inorg_ concentrations in the liver of planktivorous fish (herring and sprat) were higher (73 and 55% of THg, respectively) than those found in their muscle tissues (20 and 47% of THg, respectively). In contrast to CH_3_Hg^+^, inorganic mercury was mostly concentrated in the liver, where the process of detoxification of CH_3_Hg^+^ into Hg_inorg_ occurs. As expected, piscivorous and omnivorous fish specimens had higher CH_3_Hg^+^ levels, while planktivorous fish had higher Hg_inorg_ levels_._

The liver:muscle ratio (L:M) coefficient also confirmed the concentration gradations presented above for both forms of mercury in the fish species assayed (Table 4). The L:M was calculated considering the predominance of inorganic mercury (Hg_inorg_ = THg – CH_3_Hg^+^) in hepatic tissue (L) and of CH_3_Hg^+^ in muscle (M) (Cizdziel et al. 2003).

L:M values above 1 indicated increasing Hg_inorg_ levels in hepatic tissue, while those below 1 indicated increasing CH_3_Hg^+^ levels in muscle. L:M values above 1 for herring (2.77) and of about 1 (0.94) for sprat indicated increasing Hg_inorg_ levels in hepatic tissue, compared to CH_3_Hg^+^ in muscles. The L:M value below 1 observed for cod (0.12) and eel (0.20) could be explained by higher levels in muscle of CH_3_Hg^+^, the assimilation of which stemmed from the piscivorous and omnivorous habits of these species. In turn, the comparatively higher L:M ratios in invertivore species (herring and sprat; zoo- and phytoplanktivorous) were probably associated with prey from lower trophic levels that were more likely to have reduced CH_3_Hg^+^ concentrations in their tissues (Cremona et al. 2008, 2009). In other words, the distribution of Hg_inorg_ and CH_3_Hg^+^ is related to fish species and their prey. Finally, food is a relevant ecological factor that explains the distribution of the chemical forms of mercury among fish tissues.

## Conclusions

The following conclusions were drawn from the results of the study:

- the bioaccumulation of CH_3_Hg^+^ and Hg_inorg_ in fish varied and depended on the tissues and organs;
- the bioaccumulation of mercury forms depended largely on the feeding habits of species;
- varied L:M coefficient (L = Hg_inorg_ in liver; M = CH_3_Hg^+^ in muscle) values for the four species assayed indicated that the assimilation of various forms of mercury and their accumulation in fish tissues and organs depended on feeding habits;
- Hg_inorg_ in fish tissues and organs come from three sources:
  - directly from the environment (water and sediment) the evidence of which was high levels of this form of mercury in gills,
  - from food, which was indicated by the concentrations of mercury in digestive tracts and liver,
  - from the process of the demethylation of CH_3_Hg^+^ to Hg_inorg_, which occurs in fish in the internal organs of the liver, spleen, and heart.
